# Quantitative analysis of communication dynamics in agile software teams through multimodal analytics

**DOI:** 10.1038/s41598-025-91328-x

**Published:** 2025-03-22

**Authors:** Diego Miranda, Rene Noel, Jaime Godoy, Carlos Escobedo, Cristian Cechinel, Roberto Munoz

**Affiliations:** 1 Escuela de Ingeniería Informática, University of Valparaíso, Valparaiso, Chile; 2 Centro de Ciências, Tecnologias e Saúde, Universidade Federal de Santa Catarina, Araranguá, Brazil

**Keywords:** Computer science, Information technology

## Abstract

Effective collaboration in agile software development depends on communication, cooperation, and coordination. While cooperation is often ensured through defined roles and expertise, coordination—how participants interact during collaborative tasks—remains difficult to evaluate objectively. Agile methods promote coordination through techniques like planning poker, yet there is limited empirical evidence of their actual impact on team communication. Multimodal Analytics (MmA), which enables the quantitative analysis of verbal, paraverbal, and non-verbal cues, offers a promising approach to address this gap. This study examines the effect of coordination techniques on team communication using MmA in a controlled experiment. A total of 72 undergraduate students formed 18 teams of four, self-organized to reflect real-life group formation. Each team performed two software effort estimation tasks, one without coordination (ad hoc) and another using planning poker. The study, designed as a controlled experiment, addresses three research questions regarding the impact of coordination on speaking and attention patterns. Audiovisual recordings were analyzed to measure two dependent variables: speaking time and attention time. For each, we calculated three metrics: total time, average time per participant, and standard deviation among participants. Speaking time was measured via speech diarization, while attention time was inferred from participants’ facial orientation during verbal interactions. Results indicate that while planning poker does not significantly alter total speaking or attention time, it fosters a more equitable distribution of speaking time. This suggests an improvement in balanced participation, an important feature of effective collaboration. The study demonstrates MmA’s utility in capturing subtle team dynamics and contributes empirical evidence on the role of coordination techniques in enhancing collaborative communication in agile environments.

## Introduction

Multimodal Analytics (MmA) is an approach that enables the quantitative measurement of various elements of communication^1–3^, covering verbal, paraverbal, and non-verbal aspects. This method provides data and visualizations that facilitate understanding the behavior of subjects evaluated in various contexts. By using MmA, it is possible to obtain a detailed view of communication components that otherwise might remain hidden or would require deep and laborious analysis of video recordings by experts. Some communicational aspects that have been addressed with MmA include the detection of body postures^4,5^, facial expressions^6–8^, and verbal interventions^2,8,9^, among others.

Communication is key to collaborative work, which is one of the cornerstones of creative multidisciplinary work. In particular, in the field of software engineering, agile methods^10^ have placed collaboration at the center of the software development process, considering it in the planning of a development sprint, in the daily evaluation of progress, in the review of results, and in the retrospective analysis of the performance of the development team^11^. However, collaboration does not spontaneously occur simply by bringing people together around a goal. The conceptualizations of the domain^12,13^ have characterized collaboration as the result of effective *communication*, *cooperation*, and *coordination*. *cooperation* refers to the contribution of the different participants from their assigned roles and their knowledge and experience. However, even having experts with assigned roles, collaboration also depends on *coordination*, that is, an agreement that defines how participants will interact in the collaborative activity. In agile methods, in particular, different coordination dynamics have been proposed, which, in theory, facilitate team collaboration. An example of these techniques is *planning poker*^14^, which defines a form of interaction for the participants of a development team to estimate the effort for the development of user stories, representing the features that the software should have. However, there is no scientific evidence on the effectiveness of these interaction dynamics, as their effect must be evaluated on a third component that, so far, has been elusive to measure quantitatively: *communication*.

Communication is related to other concepts and skills, such as collaboration and cooperation, which play a crucial role in building strong interpersonal relationships and enhancing the efficiency and effectiveness of teamwork^15,16^. Additionally, these competencies are recognized as skills of the twenty-first century^17–19^ and are highly sought after by employers^20,21^. However, effective communication is not an easy skill to achieve, as it does not occur merely when a group of people meets to talk to them. Instead, it requires that participating members are committed to the progress and achievement of the team, implying both cognitive and motivational dedication^22^. Studies have shown that within a classroom context, students do not know how to communicate effectively, especially at the beginning of these sessions^23,24^, making it necessary to support students in improving their communication skills. The presence of a teacher or facilitator is required to guide learning and interactions, allowing students to articulate important ideas and participate in more meaningful exchanges^23,25^.

It is noteworthy that non-verbal communication plays an integral role in these interactions, and it is crucial to acknowledge that it often gets overlooked, especially during teamwork. Nonverbal communication includes gestures, facial expressions, body language, and tone of voice, among others^26^. These elements can convey a wealth of information and often complement or even contradict our words^27,28^. However, in a team working environment, nonverbal communication can be difficult to perceive and easy to ignore^29^.

Considering how important and traditionally difficult it has been to objectively evaluate communication, MmA emerges as a possibility to conduct quantitative collaboration studies. According to the conceptualization of collaboration^12^, the application of MmA allows the combination of nonverbal and paraverbal elements to evidence changes in communication produced by different coordination agreements in teams while keeping the definitions of roles and responsibilities (cooperation) stable. The purpose of this study is to evaluate the effect of using a coordination dynamic on the time and attention of participants in a collaborative activity, measured with MmA techniques. The context in which the study is conducted involves agile software development teams that estimate the effort of a group of user stories, which must be achieved through a collaborative agreement. Each team first performs an effort estimation without a defined form of coordination and then, using planning poker, in two different but similar problems, maintaining their participants’ roles. During both sessions, audio and video are recorded and analyzed with MmA techniques to measure speaking time and attention while speaking. Speaking time is measured using diarization techniques to discriminate the oral interventions made by each participant, and attention is measured using the orientation of the face among participants during such interventions. After the experimental sessions, charts and visualizations consolidating the MmA measurements were presented to domain experts, who generated hypotheses from their observations. In recent years, significant advancements have been made in the field of multimodal analytics (MmA), particularly in the analysis of non-verbal communication. Studies such as Grover et al.^4^ and Cukurova et al.^5^ have demonstrated the utility of MmA in evaluating collaboration dynamics through the analysis of body postures and spoken interactions during collaborative tasks, while Kim et al.^7^ introduced transformer-based fusion networks for facial expression recognition, highlighting the potential of integrating audio-visual data to enhance emotion detection. This emerging field integrates multiple modalities, such as audio and video, using machine learning techniques to automate the assessment of complex constructs^30^. Research has demonstrated its effectiveness in various contexts, such as job interviews, where facial expressions, speech, and prosodic information can be analyzed to provide feedback on engagement, speaking rate, and eye contact^31^, and healthcare simulations, where spatial patterns and voice detection data model team communication and extract non-verbal events like total speaking time and overlapped speech^32^. Despite these advancements, the application of MmA to agile software development teams, specifically through techniques like planning poker, remains underexplored.

In this study, we propose a novel set of metrics and an experimental setup using MmA to quantitatively measure communication dynamics within agile software development teams. Our approach focuses on capturing and analyzing nonverbal communication elements, such as speaking time and attention distribution, to assess the impact of the planning poker coordination technique on team collaboration. The results demonstrate the efficacy of our proposed method in providing objective insights into team communication patterns, thereby contributing to the field of collaborative work in co-location environments. Our study builds on these foundations by specifically focusing on the use of MmA to measure communication dynamics within agile software development teams, particularly through the planning poker technique. While our work is not the first to employ MmA for non-verbal communication measurement, it is novel in its application to this specific context and in its detailed analysis of speaking time and attention distribution among team members.

## Methods

Our research aims to study the effect of a coordination dynamic on collaboration, leveraging the potential of MmA to measure nonverbal and paraverbal aspects of communication. It is situated in the specific context of collaborative activities for agile software development^33,34^, particularly the use of planning poker^35^ for sprint planning. We chose planning poker due to its widespread use and recognized importance in agile software development teams for effort estimation. This technique facilitates structured discussions, allowing all team members to contribute their estimates and reasoning. We define the study according to the guidelines presented by Wohlin et al.^36^.

Analyzing the planning poker technique with the purpose of evaluating its effect on non-verbal communication from the point of view of the researcher in the context of undergraduate students performing a co-located collaborative activity.

The research questions for the study are two, detailed below along with the respective null hypotheses.

- RQ1: What is the effect of using poker planning on the speaking time of the participants in the collaborative activity? The associated null hypothesis is *H*_0_*st*: planning poker has no effect on the speaking time of participants.
- RQ2: What is the effect of using poker planning on the attention time of the participants in the collaborative activity? The associated null hypothesis is *H*_0_*at*: planning poker has no effect on the attention time of participants.

For the first two research questions, which require measuring speaking time and attention time, the application of the MmA approach is fundamental. For this reason, the following subsection will dive into how data is collected and processed to operationalize these variables, then continue with the design of the experimental activity and data analysis.

### Data collection

For this study, data collection was carried out using a multimodal methodology, focused on capturing participant interactions through audio and video recordings. Advanced technologies were utilized to accurately capture the dynamics and interactions within the teams. Sessions were recorded using a Kandao Meeting Pro camera system, which includes a high-definition camera with the capability of recording in 360° panorama, ensuring a complete view at the center of the activity. The audio and video recordings were synchronized at the start of each session to ensure accurate alignment of the extracted features. Although the audio and video data were processed independently, we correlated the non-verbal features with the verbal interventions by matching the timestamps from the diarization process with the corresponding video frames. This approach allowed us to accurately align speaking time and attention direction, ensuring that the analysis of non-verbal communication was consistent with the verbal interactions.

#### Audio data

The audio analysis focused on understanding and quantifying the verbal interaction among participants, a critical component for assessing communication dynamics in collaborative environments^37^. By utilizing advanced voice recognition technologies, we aimed to extract accurate data on speaking time and participant diarization.

The central tool in this process was WhisperX^38^, an advanced, open-source version of the renowned Whisper voice recognition system^39^. WhisperX is distinguished by its temporal precision, offering word-level segmentation through vocadetection of vocal activity forced phoneme alignment. This capability significantly enhances the identification and allocation of audio segments to the corresponding speakers, a process known as diarization. Diarization is crucial for determining who is speaking and when, allowing for a detailed analysis of the group’s communication dynamics.

To ensure the quality and accuracy of the data, all audio files were converted to WAV format and processed using the Large-v3 model, which presents a lower word error rate. The resulting transcriptions were stored in JSON format, facilitating their analysis and integration with visual data.

Manual review of each file was a critical step in ensuring the accuracy of the information. This meticulous process involved verifying timestamps, correcting speaker misidentification, and removing incorrectly assigned audio segments. This rigorous approach ensured that the analyzed data reliably reflected verbal interactions during the sessions, minimizing distortions and providing a solid foundation for further analysis.

#### Video data

##### Participant detection and tracking

**Fig. 1:**
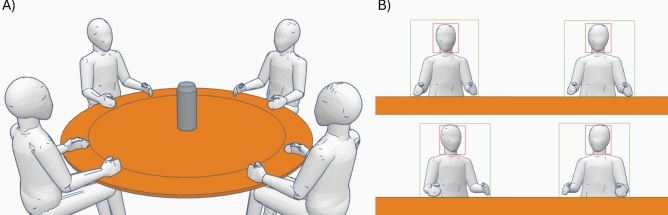
**(A**) Example of a group activity viewed from the outside. (**B**) Example of a group activity viewed with the 360-degree camera. The bounding box of the participant is indicated in green. The bounding box for the participant’s face is indicated in red.

Initially, we applied YOLOv8^40^ to accurately identify each participant in the video recordings. This real-time object detection model excels in recognizing and labeling individuals quickly, generating bounding boxes around each one, marked in green in the visualization of Fig. 1B. Subsequently, we used DeepSORT^41^, through the Python library *deep_sort_realtime*, to track the movement of the participants throughout the session. This combination of detection and tracking ensures the precise correlation of individuals with their specific actions.

##### Facial landmark detection

**Fig. 2:**
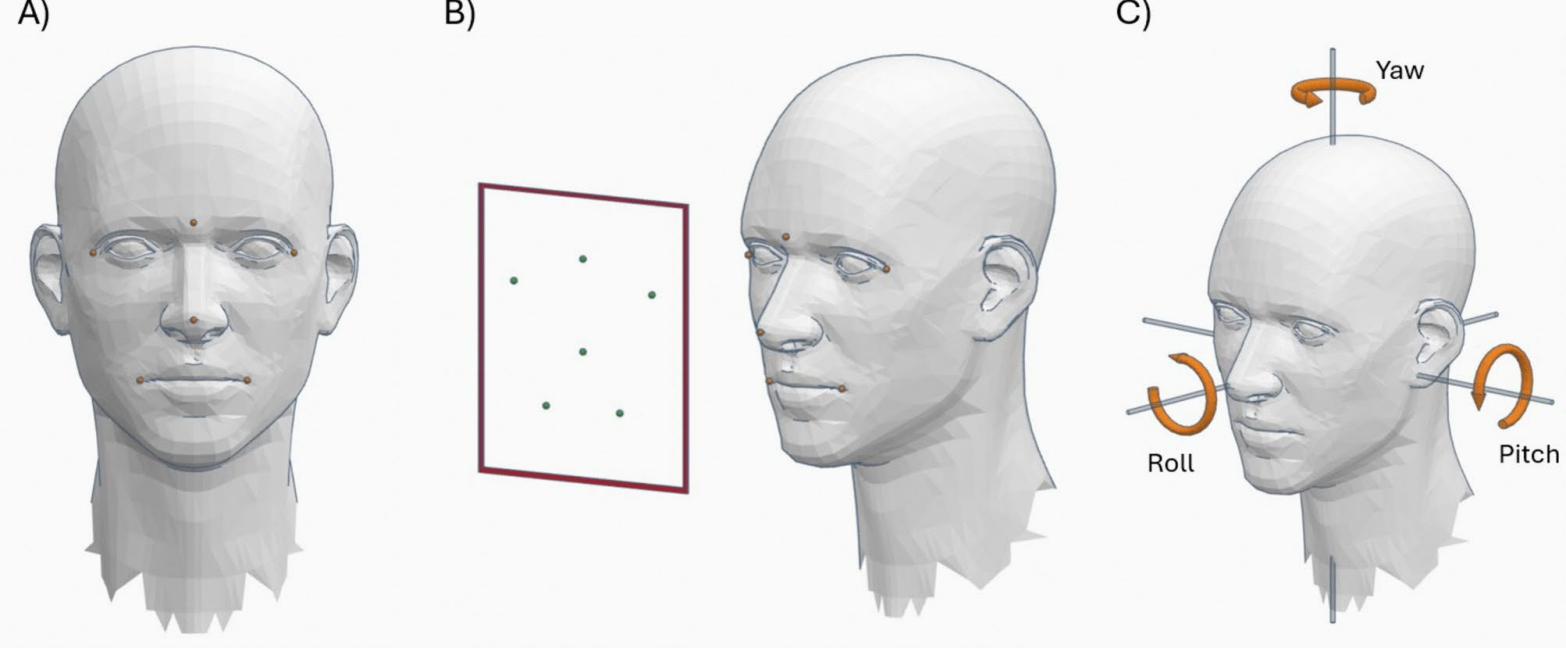
**(A**) Example of the facial points used to estimate its direction. (**B**) Example of 2D points relative to the 3D points of the face. (**C**) Diagram of the rotation axes *pitch*, *yaw* y *roll*.

With the participants clearly identified, we used facial landmarks detected by MediaPipe^42^ to detect the face and capture the fine details of non-verbal communication (bounding box in red, from Fig. 1B). The landmarks are 1, 9, 57, 130, 287, and 359 according to the MediaPipe documentation, illustrated in Fig. 2A. These points are crucial for assessing the orientation of the face and, therefore, the attention, interest, and participation of subjects during group interaction.

##### Accurate face direction estimation

The direction of the face is estimated using the solvePnP (Perspective-n-Point) function of OpenCV^43^, which calculates the three-dimensional orientation of the face based on known facial landmarks. This process involves transforming these points from a 3D model to their corresponding location in the 2D image (see Fig. 2B). From this function, we obtain a rotation vector that is converted into a rotation matrix to determine the face orientation axes: *pitch*, *yaw*, and *roll* (see Fig. 2C).

By obtaining the face orientation, having a camera with panoramic vision, and all participants at the same distance, we can estimate towards which other participant their face is pointing. This is done considering that the camera has 45 and 360 (2 images of 180)° of vision on the vertical and horizontal axes, respectively, and the participants are at the same distance from the camera. We convert the point of the nose (1) into a vector $\overrightarrow {v}$, whose angle represents the location of the participants’ faces with respect to the camera’s coordinate axis.

$$\overrightarrow {v} = r(cos\left( a \right)x + sin\left( a \right)y + tan\left( b \right)z)$$

*a* y *b* are the vertical and horizontal angles of the nose, respectively, and *r* is the distance of the participants from the camera. Having obtained the vector of the location of each participant and the direction of the face, the focus vector *v⃗*_*f*_ that indicates where a participant is directing their face, from the camera’s perspective, is calculated. This calculation is based on the following formulas:

$$lx = r + r\cdot\cos (2 \cdot yaw)$$

$$ly=r* \text{sin}(2*yaw)$$

$$h= \sqrt{{lx}^{2}+{ly}^{2}}$$

$$\vec{v} = \left[ {\begin{array}{*{20}c} { - \vec{v}_{x} *{\text{cos}}\left( {2*yaw} \right) - ( - \vec{v}_{y} *{\text{sin}}\left( {2*yaw} \right))} \\ { - \vec{v}_{x} *{\text{sin}}\left( {2*yaw} \right) - ( - \vec{v}_{y} *{\text{cos}}\left( {2*yaw} \right))} \\ { - \vec{v}_{z} + lz} \\ \end{array} } \right]$$

##### Detailed analysis of attention

Finally, having obtained the focus vector of all participants, the reverse process is conducted to obtain the angle with respect to the camera. At this point, to determine whom a participant is observing, 2 thresholds were established. The first threshold (vertical axis) corresponds to whether the person is holding their head up or looking at documents. This threshold was determined to be 15° (out of a total of 45 degrees), which means that if the vertical angle is below 15°, the participant is reading; otherwise, they could be observing another participant. The second threshold (horizontal axis) was determined to be 25%, which means that if participant *A* has participants *B*, *C*, and *D* in front, to the right, and to the left, respectively, the degree distance regarding the camera from participant *B* to *C* and *D* will be calculated, then 25% of this distance is calculated.

$$u_{r} = \left( {\left| {angle\left( C \right)} \right| - \left| {angle\left( B \right)} \right|} \right) * 0.{25}$$

$$u_{l} = \left( {\left| {angle\left( B \right)} \right| - \left| {angle\left( D \right)} \right|} \right) * 0.{25}$$

Let *u*_*r*_ be the threshold to the right of participant *B* and *u*_*l*_ the threshold to the left of the participant. Then, if the horizontal angle of the focus vector of *A* is less than the threshold *u*_*r*_, it is determined that *A* is paying attention to *C*. If the horizontal angle is greater than the threshold *u*_*l*_, it is determined that *A* is paying attention to *D*. When the horizontal angle is between the threshold *u*_*r*_ and *u*_*l*_, it is determined that *A* is paying attention to *B*.

With the aforementioned analysis, it is possible to determine if, in a specific frame of the video, a participant is paying attention to another, their faces falling within the previously described thresholds. By summing up the frames, it is possible to calculate the amount of attention time each participant has given. To determine whether attention is being paid during a verbal intervention, only the frames whose timestamp falls within the range of verbal interventions determined for the participant receiving the attention are considered. The times of verbal interventions are determined by diarization, as explained earlier in the audio detection subsection.

The final results are stored in CSV format, documenting the number of frames in which a participant directs their attention towards another.

#### Validation methodology

To validate the accuracy of our proposed measurements, we employed a multi-step validation process, ensuring robustness and reliability in our analysis of communication dynamics. This approach integrated advanced AI validation, pilot study comparisons, mathematical verification, and iterative review sessions by our research team.

- Artificial intelligence validation: The AI models utilized in our study, specifically WhisperX for audio diarization, YOLOv8 for participant detection and MediaPipe to estimate the key points of the face, were selected due to their proven efficacy in previous research. These models have been rigorously tested across various scenarios, demonstrating high precision in detecting and analyzing verbal and non-verbal communication. This foundation provided a solid basis for our confidence in their application.
- Pilot study: We conducted a pilot study with a subset of our participants to validate the performance of our models in a real-world setting. This pilot involved detailed manual annotations of a sample of the audio and video data by our research team. These annotations were then compared with the results produced by our models to assess their accuracy. The pilot study was designed to be equivalent in terms of the number of participants, duration, and interactions to ensure consistency and reliability of the results.
- Mathematical verification: The algorithms used to estimate speaking time and attention were subjected to mathematical validation. We created synthetic datasets with known parameters to test the algorithms under controlled conditions, confirming their accuracy in these scenarios.
- Iterative review: The results of our analyses were reviewed in multiple sessions by the research team. We examined the outputs of the models on existing recordings, identified any discrepancies, and refined the algorithms iteratively. This process included both qualitative and quantitative assessments to ensure the models’ outputs aligned with human observations.

These validation steps provide a robust framework to ensure the correctness of our proposed measurements, even in the absence of extensive human annotation for the entire dataset. By leveraging AI validation, pilot studies, mathematical verification, and iterative reviews, we ensured that our measurements accurately reflected the communication dynamics observed in the recorded sessions.

### Experimental design

#### Factors and variables

The factor under study is the coordination technique, which has two levels: ad hoc, or without prior coordination instructions, and planning poker. The dependent variables associated with the three research questions and the metrics that operationalize them are described below.

- Speaking time: Refers to the time that each participant speaks during the activity. Three metrics were calculated: total speaking time for all participants, average speaking time among participants, and standard deviation of speaking time for each participant.
- Attention time: Refers to the attention time of each participant during verbal interactions. The direction of attention was analyzed based on the orientation of the face, determining toward which other participants this attention was directed and at what moments. With these data, three metrics were calculated: the total attention time of all participants, the average attention time of each participant, and the standard deviation of attention time for each participant.

The subject of the study is a collaborative group composed of four participants. Each group participates in two experimental activities; in the first, they collaborate without a coordination technique (Activity A), and in the second, they use planning poker (Activity B), providing a uniform comparative basis. Teams have a maximum of 10 mins to reach an agreement. The agreement consists of rating the complexity of developing a user story that describes a software functionality in a 2-week sprint. User stories, defined by their complexity level, are classified using a five-level Likert scale. This scale ranges from *Very Simple*, where the problem requires minimal effort and can be quickly completed within the Sprint, to *Impossible*, indicating that the user story exceeds the team’s capacity to be developed within the assigned Sprint time. The intermediate levels, *Simple*, *Medium*, and *Complicated*, offer varying degrees of difficulty and possibilities for including additional work in the Sprint, thus providing a realistic spectrum of challenges that software development teams regularly face.

The experiment considers keeping certain independent variables constant throughout the experiment to block their effect. In particular, the composition of the team, the type and complexity of the estimated user stories, and the physical and technological environment in which the sessions are conducted, to ensure the validity and reliability of the results. Additionally, each participant has a unique role within the team from which they must cooperate, which can be Backend, Frontend, UI/UX, and data persistence. This allows isolating the effect of introducing planning poker on the interest variables.

During the experiments, participants were positioned around a circular table, facilitating precise detection and recording of interventions and behaviors, such as visual focus and attention to other members. Each participant received a detailed introduction to the problem to be solved and a diagram of the related architecture, ensuring equality of information in the discussions.

### Participant selection

During the months of August and November 2023, a public call was extended to students of the School of Computer Engineering at the Universidad de Valparaíso to voluntarily participate in recording sessions. Students interested in participating were required to have previously completed the course on Fundamentals of Software Engineering. This requirement was established to ensure that all participants had a common basic knowledge related to software development and agile methodologies.

We received approval from the Institutional Bioethics Committee for Research on Human Subjects of the Universidad de Valparaíso (protocol code CEC-UV 236-21). Participants gave informed consent before joining the study, ensuring their understanding and agreement with the terms of their participation. No financial compensation was offered for their participation, highlighting the voluntary nature and personal interest in contributing to the advancement of research in software engineering and team dynamics. Additionally, the study was conducted in accordance with the principles of the Declaration of Helsinki, ensuring that the research adheres to the ethical standards for research involving human subjects.

Group formation was based on the participants’ preferences, who had the freedom to choose whether they wanted to collaborate with people they already knew or with new peers. This flexibility allowed for diversity in the composition of the teams, reflecting real-life team formation situations in educational and professional environments. Each group consisted of four students, achieving a total participation of 72 students, which resulted in the formation of 18 different teams.

To document and analyze the development of the activities and the dynamics of the teams, detailed information on the gender distribution of the participants, the time dedicated to each activity, and the level of agreement reached by each team was collected. This information is summarized in Table 1, which provides an overview of the team dynamics and the outcomes of the activities under the different experimental conditions.

**Table 1:** Information for each group after the activity is completed.

Group	Number of participants		Time spent (min)		Level of complexity	
	Male	Female	Without coordination	With coordination	Without coordination	With coordination
1	3	1	8:10	9:30	Medium	Complicated
2	4	0	10:00	9:30	Medium	Complicated
3	4	0	7:34	10:00	Simple	Simple
4	4	0	7:30	10:00	Medium	Complicated
5*	4	0	9:46	9:45	Medium	Medium
6	2	2	7:20	7:15	Simple	Simple
7	1	3	3:40	4:27	Simple	Complicated
8	4	0	7:47	3:30	Impossible	Simple
9	4	0	10:00	5:00	No agreement	Simple
10**	4	0	9:35	9:10	Complicated	Medium
11	4	0	9:32	9:49	Complicated	Complicated
12	2	2	10:00	6:15	Very simple	No agreement
13*	3	1	4:46	8:11	Medium	Impossible
14	3	1	9:10	6:00	Complicated	Complicated
15	4	0	10:00	7:09	Medium	Very simple
16	3	1	9:40	9:52	Medium	Medium
17	3	1	4:50	6:00	Medium	Simple
18	4	0	5:38	7:55	Simple	Medium

An * indicates that participants were removed from the sample for not following the instructions of the experiment. A ** indicates that the data are considered outliers.

Below are the detailed results for each experimental variable.

### Data analysis

To answer research questions, an analysis was performed using statistical and computational tools to process and analyze the data sets obtained from audio and video recordings, allowing an objective and quantitative interpretation of communication and collaboration dynamics within teams. Statistical analysis was carried out following these steps:

- Data Inspection: Box plots are created for each measurement to study the presence of outliers in the collected data, as these extreme values can significantly influence the underlying assumptions of many statistical tests. To quantitatively identify outliers, the interquartile range (IQR) method is used. This method defines outliers as points below *Q*_1_–1.5 or above *Q*_3_–1.5 × *IQR*, where *Q*_1_ *Q*_3_ are the first and third quartiles, respectively, and *IQR* is the interquartile range (*Q*_3_–*Q*_1_).
- Verification of prerequisites for t-test: A comparison between the conditions with and without predefined coordination was performed to evaluate the impact of planning poker on the communication and collaboration of the teams. The Shapiro–Wilk test^44^ was used to verify the normality of the data distribution for each measurement, and the Levene test^45^ was used to verify the homogeneity of variances.
- Application of t-test: After verifying the necessary prerequisites, the t-test is applied to compare the difference between the measurements with and without coordination. Its statistical significance is verified if the p-value is less than or equal to 0.05.
- Calculation of effect size (Cohen’s *d*): To complement the analysis with the t-test and gain a deeper understanding of the actual impact of the intervention, we calculate the effect size using Cohen’s *d*. This indicator allows us to quantify the magnitude of the difference between the coordination and no-coordination groups, providing a measure of the practical relevance of the results. Cohen’s *d* is calculated as the difference between the means of the two groups divided by the combined standard deviation of the groups. A *d* value of 0.2 is considered a small effect, 0.5 a medium effect, and 0.8 or more, a large effect. This calculation helps us interpret the statistically significant differences observed and have practical relevance in the study context.

#### Threats to validity analysis

Regarding threats to the validity of this study, it is important to mention several aspects that could influence the interpretation and generalization of the results. One threat is the learning effect, which may arise when participants are exposed to both experimental conditions (with and without planning poker). This threat is associated with the risk that the experience gained in the first session influences performance in the second, regardless of the coordination technique used. To mitigate this threat, the experiment’s design included changing the user stories between sessions and alternating the conditions for each group. However, participants were not informed of the specific aspects being measured, which reduces the possibility that they intentionally modified their behavior to adapt to the perceived expectations of the study.

Another potentially relevant threat is participant selection. Since participation in the study was voluntary and targeted at a specific group of computer engineering students with a certain knowledge of agile methodologies, the results may only be generalizable to some contexts of agile software development or teams with different experience levels. Although the nonrandom selection could limit the generalization of the findings, the diversity in team formation, based on participants’ preference to collaborate with known or new peers, attempts to reflect varied team dynamics found in educational and professional environments.

Finally, another domain of threats to validity concerns the definition and measurement of variables. Although this study sought to apply a rigorous and multimodal approach to capture communication and attention dynamics in teams, caution is required in interpreting the collected data. It is possible that the metrics of speaking time and attention do not fully capture the complexity of these interactions in a team-working environment. Future research could incorporate additional measures, such as analysis of communication content or evaluation of the quality of work produced, to better understand how coordination techniques influence effective collaboration.

## Results

A total of 18 groups and 72 participants completed the experimental activities. The results of the gender distribution of the participants in each group, the duration of the activity and the outcome of the complexity estimation are shown in Table 1.

After data collection, Groups 5 and 13 were discarded because it was verified during the activity that the participants did not follow the established procedure.

### Speaking time

**Fig. 3:**
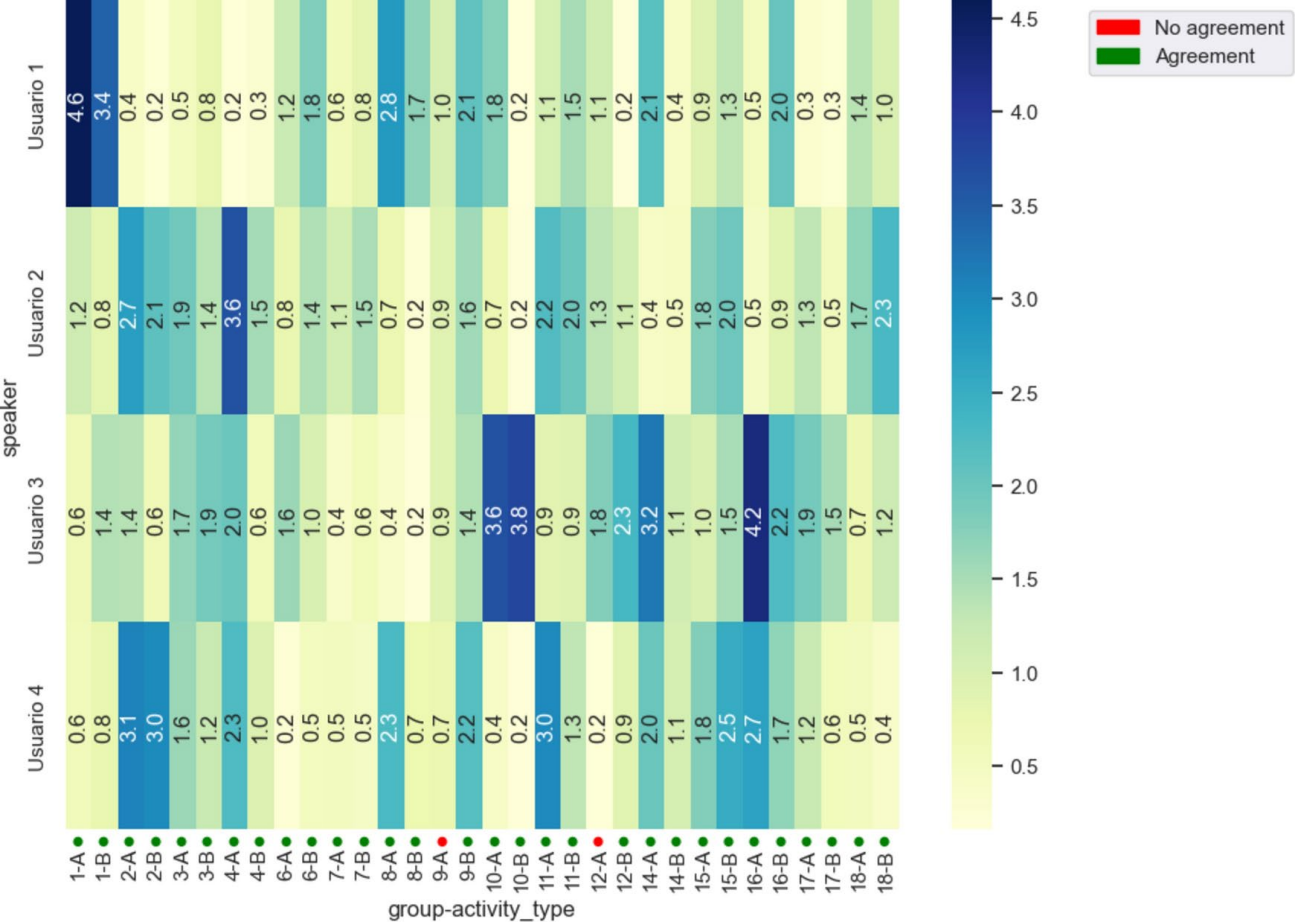
Heatmap of total speaking time in minutes per group and activity.

The application of MmA allowed for the rapid generation of visualizations to guide the analysis of the produced data. In Fig. 3, a heatmap is shown comparing, for each group, the speaking time in their session without coordination (Activity A) and with coordination (Activity B). Through simple inspection, it is possible to hypothesize that the “heat” of the groups tends to moderate in Activity B compared to Activity A.

**Fig. 4:**
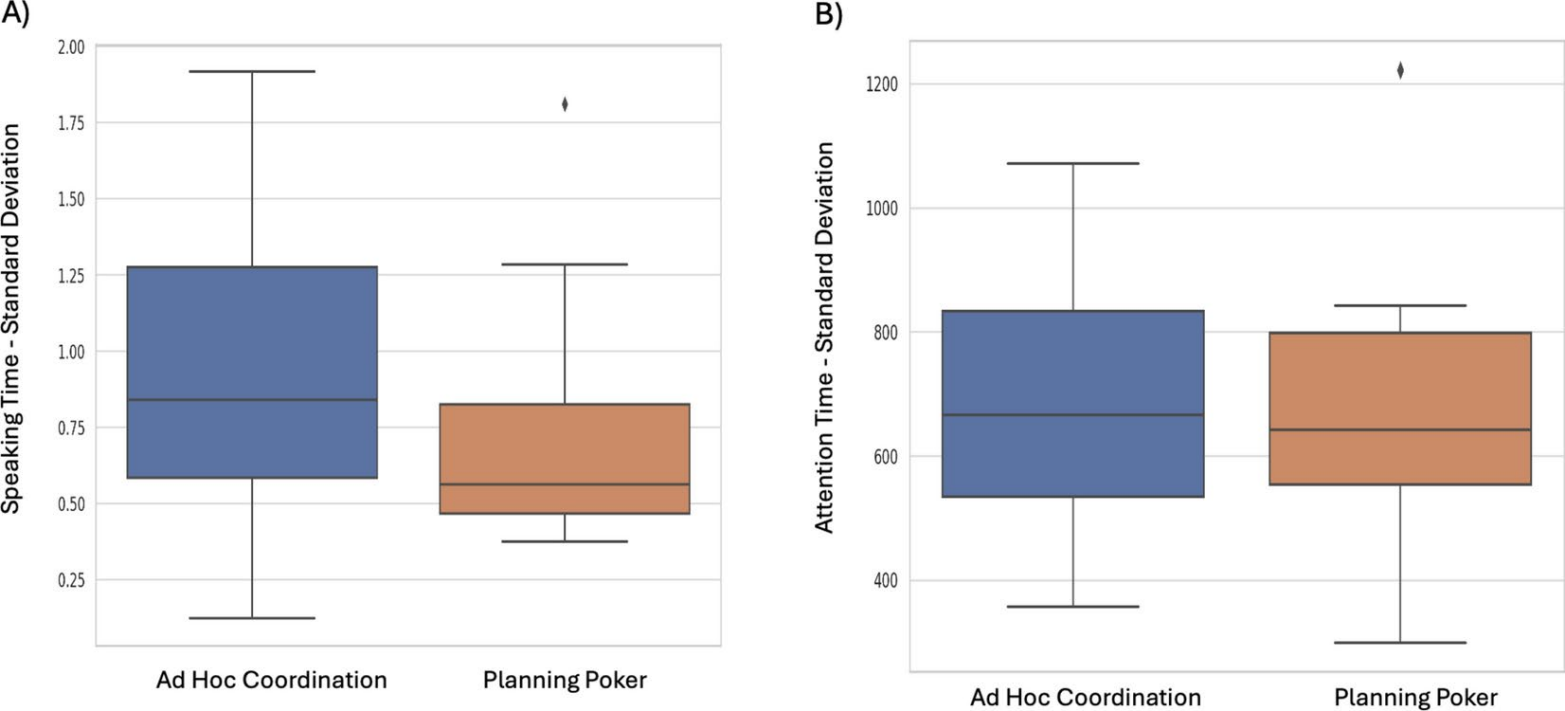
Boxplots for the Standard Deviation of Speaking Time (**A**) and Attention Time (**B**). (**A**) Shows the distribution of speaking time standard deviation among participants for sessions with ad hoc coordination and planning poker. The results indicate a reduction in the standard deviation of speaking time with planning poker, suggesting a more equitable distribution. (**B**) Displays the distribution of attention time standard deviation, highlighting no significant difference between ad hoc coordination and planning poker, implying that planning poker primarily impacts speaking time distribution rather than attention patterns. These findings suggest that other dynamics, beyond the coordination technique, influence attention distribution.

Outliers for speaking time across the three metrics: Total Speaking Time (TST), Average Speaking Time (AST), and Speaking Time Standard Deviation (STSD), for activities without coordination and with coordination, were reviewed. As seen in Fig. 4A), the boxplot for STSD shows an outlier. After conducting the interquartile range analysis to identify the outlier and verifying in the video recording that group 10 had not followed the procedure, it was removed from the analysis.

The normality and homogeneity of variance tests were passed for all measurements, which allowed the application of the t-test to evaluate significant differences. The statistics of the tests are detailed in Table 2.

**Table 2:** Speaking time statistics.

Variable	p-value (Test t)	Shapiro–Wilk A	Shapiro–Wilk B	Levene’s test
TPH	0.139930	0.378962	0.237367	0.560153
TTH	0.139930	0.378962	0.237367	0.560153
DEH	0.050868	0.495974	0.002419	0.059034

The results show that while the p-value of the t-test does not reach 0.05 for any of the measurements, for the Speaking Time Standard Deviation (STSD), its value is very close to statistical significance (*p* = 0.560153). The effect size of this difference is large (Cohen’s *d* = 0.610), suggesting that the differences can be perceived in practice.

The interpretation of this result is that the coordination technique allows subjects who spoke less without defined coordination to now speak more, and those who spoke more, now speak less. This would democratize the use of speaking time during collaborative activities.

With this, the null hypothesis *H*_0_*st* for the standard deviation of the participants’ speaking time is rejected.

### Attention time

**Fig. 5:**
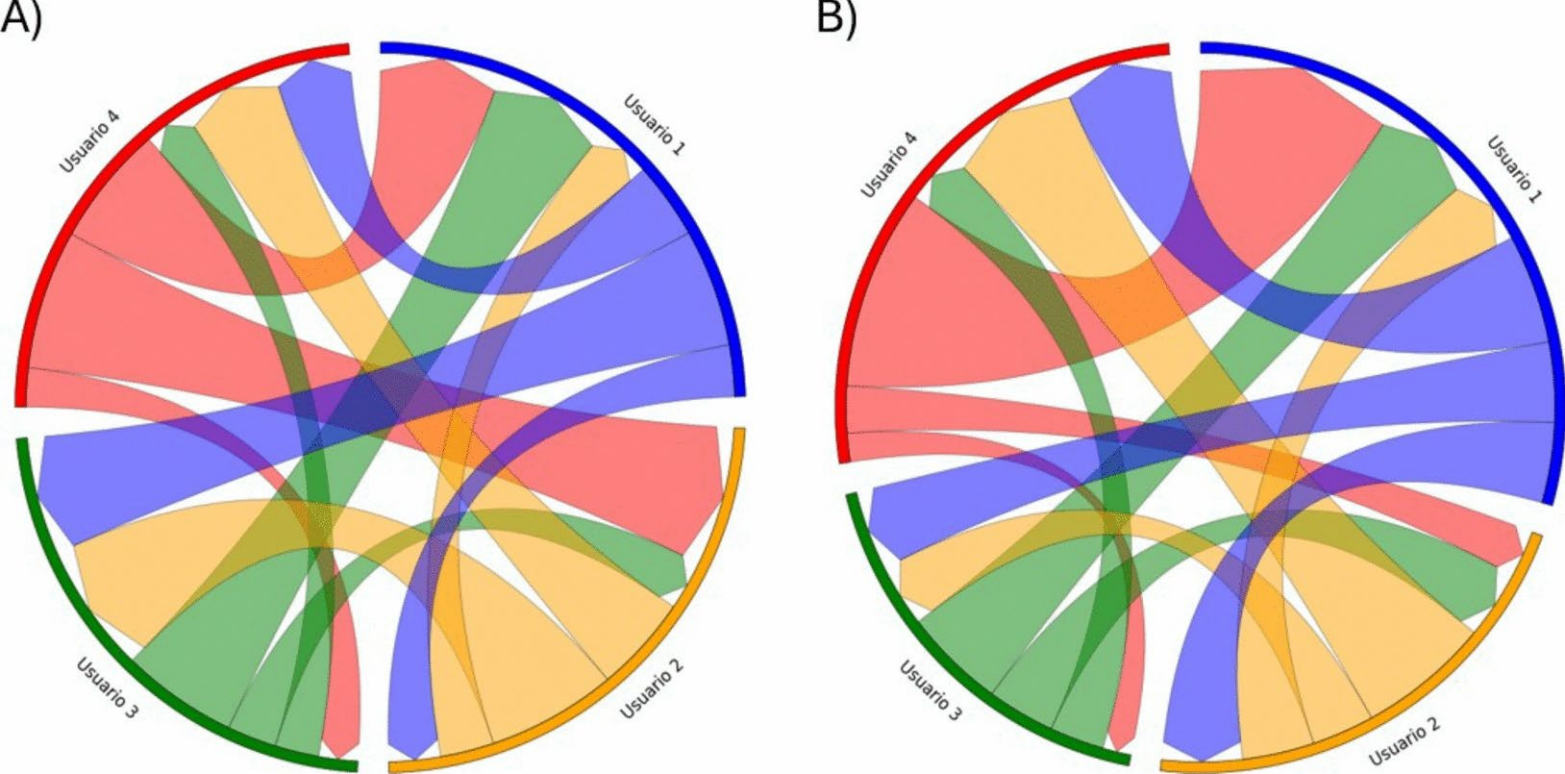
Attention time chart for Group 11, Activity A and Activity B. The length of the arc segment represents the speaking time of each participant, the width of the arrows represents the attention time during speaking, and the direction of the arrow indicates the user’s attention to others (arrows from the participant) and the time the participant receives attention from others (arrows towards the participant).

To explore the results of attention times, visualizations were developed for each group, like the one shown in Fig. 5, which account for the amount of time each participant pays attention to others while they are speaking, as well as the attention they receive while the subject speaks. The visualizations were compared with the video recording of each activity, finding minor errors in the automatic detection in the identification of users, which were corrected before the analysis of results. At first glance, no distinct attention patterns between the two activities each group participated in were identified, such as subjects who initially did not receive attention and then did, for example.

Outliers for the attention time across the three metrics: Total Attention Time (TAT), Average Attention Time (AAT), and Attention Time Standard Deviation (ATSD), for activities without coordination and with coordination, were reviewed. As seen in Fig. 4B), the boxplot for ATSD shows an outlier. After conducting the interquartile range analysis to identify the outlier, it corresponds to Group 10, which was identified and excluded in the previous subsection.

Normality and homogeneity of variance tests were passed for all measurements, allowing the application of the t test to evaluate significant differences. The statistics for the tests are detailed in Table 3.

**Table 3:** Attention time statistics.

Variable	p-value (Test t)	Shapiro–Wilk A	Shapiro–Wilk B	Levene’s test
TPA	0.848904	0.980390	0.258090	0.953797
TTA	0.848904	0.980390	0.258089	0.953797
DEA	0.346346	0.272863	0.562062	0.329771

The results of the Attention Time statistical tests indicate that no statistically significant differences were found between conditions with and without the use of the coordination technique for any of the metrics related to Attention Time. The p-values for the t-tests of these metrics exceed the statistical significance threshold of 0.05, implying that there is not enough evidence to reject the null hypotheses associated with these measures.

Specifically, for the ATSD, even after a detailed inspection of outliers and the exclusion of Group 10, the resulting p-value of the t-test (0.346346) remains far from the threshold of 0.05, indicating that there is no significant difference in the variability of attention time between the two coordination conditions. This suggests that the use of planning poker did not significantly affect the way participants distributed their attention during collaborative activities.

## Discussion

In relation to the first research question (RQ1) which explores the impact of employing the technique known as planning poker on the participation time of those involved, it is observed that, despite there being no statistically significant differences regarding the total intervention time, the standard deviation of speaking time exhibits a considerable decrease when this coordination methodology is implemented. This finding suggests that the use of the planning poker technique encourages a more equitable distribution of speaking time among participants, thereby contributing to greater equality in the allocation of intervention time during collaborative activities. Consequently, the null hypothesis *H*_0_*st* associated with the standard deviation of speaking time is rejected, confirming that planning poker significantly affects this variable.

Regarding the second research question (RQ2), which examines the impact of using planning poker on participants’ attention time, the results reveal that no statistically significant differences were recorded in any of the metrics associated with attention time. This finding suggests that, although the planning poker methodology may influence the distribution of participation time by those involved, it does not significantly affect the way they pay attention during activities. Therefore, the null hypothesis *H*_0_*at* is accepted, indicating that planning poker does not have a significant effect on the attention time of the participants.

The difference in results between speaking time and attention time could be explained by the specific nature of each type of interaction. While planning poker seems to facilitate a more equitable distribution of speaking time, possibly due to its structure that grants explicit turns for participation, the attention participants pay does not seem to be directly influenced by this coordination mechanism. This could be because attention is more susceptible to individual factors and group dynamics that are not simply altered by changing the way discussion is organized, such as participants’ previous experience working together, their level of interaction, and team size, as reported by studies on team participation^46^.

These findings are consistent with previous work that has explored how different collaboration techniques affect team dynamics in agile environments, suggesting that while some practices may improve specific aspects of collaboration, their impact can vary depending on the team characteristics and context. In the work by Haugen^47^, an empirical study is presented on the impact of using planning poker for user story estimation by a team using the Extreme Programming (XP) methodology. The comparison between an unstructured estimation process and the semi-structured use of planning poker revealed overall improvements in team performance, although an increase in estimation error was observed in extreme cases. The same case for the work by Poženel et al.^48^, whose goal was to evaluate the effectiveness of effort estimation methods in software development. University students volunteered, who were not informed in detail about the study’s objectives to prevent them from manipulating the results. It was found that some agile methods, one of them planning poker, are more accurate and efficient in estimating the effort to complete user stories in a project.

Regarding effort estimation in agile methods, this work pays attention to factors not previously measured: evidence of the effects of estimation methods is usually measured based on the accuracy of the estimates^49^, however, the effective and equitable participation of team members had been unexplored until now. In this sense, the implications of this MmA application study to collaborative work transcend the agile software development context. The ability to identify differences in the spread of speaking time between participants could provide a powerful nonverbal communication indicator to assess, in real-time, situations of inequity in collaboration, which in educational contexts has been identified as a foundational component of social justice^50^.

In this research, most of the potential threats have been successfully mitigated through meticulous study design. Through the application of MmA techniques, we have been able to obtain detailed insights into the verbal, paraverbal and nonverbal aspects of communication in agile software development teams. These analyses have allowed us not only to evaluate the effectiveness of coordination techniques such as planning poker in the equitable distribution of speaking time among participants, but also to better understand how participants direct their attention during collaborative activities. Despite these advances, we recognize that the depth and reliability of our measurements can be further improved. Therefore, future work will aim to complement the multimodal analysis presented in this article with the detection of other aspects of non-verbal and verbal language, such as body posture identification and content analysis of oral interventions. The purpose of these measurements will be to contribute to the measurement and evaluation of the equitable participation of members in a collaborative work team.

In addition to planning poker, the metrics and experimental setup proposed in this study can be effectively applied to other coordination dynamics techniques used in agile software development. Techniques such as daily stand-ups, sprint retrospectives, and pair programming also involve critical communication and coordination elements that can be quantitatively measured using our MmA approach^11^. As described in Przybyłek et al.^51^, adopting collaborative games during retrospectives can enhance creativity, involvement, and communication among team members, providing comparable benefits to planning poker. Future work will aim to systematically evaluate these coordination dynamics, further validating the versatility and effectiveness of our proposed measurements in various collaborative settings.

We acknowledge that our participant pool had a strong male bias, however, it unfortunately reflects the current gender distribution in the field of computer engineering. This gender imbalance may affect communication patterns, attention distribution, and overall team dynamics, potentially introducing bias into our findings. While our study provides valuable insights into team collaboration, we encourage future research to ensure a more balanced gender representation to validate and extend our findings across diverse participant groups.

In relation to our findings on attention time, Fig. 4 indicates that planning poker does not significantly affect the total attention time. This result suggests that while planning poker promotes a more equitable distribution of speaking time, it might not directly influence the way attention is distributed among team members. This could be due to other underlying factors such as individual engagement levels, prior working relationships, and the specific context of the collaborative task. Future research should explore other coordination techniques to see if similar patterns emerge and to further investigate the factors influencing attention time in collaborative settings.

Regarding Fig. 5, the observed interaction/attention bias towards the member at the opposite side can be attributed to the physical arrangement of participants around a table, which mirrors real-world settings where individuals naturally tend to look directly across from them. While estimating attention based on face direction, this limitation can be addressed in future studies by complementing face direction data with eye-tracking technology. Eye-tracking can provide more detailed insights into where participants are looking, which would help mitigate issues related to occlusion and enhance the accuracy of attention estimation. However, current eye-tracking technology is not yet mature enough to provide reliable results in such collaborative settings, particularly due to challenges related to occlusion and the need for unobtrusive tracking methods.

Beyond the results presented in this study, the feasibility of using MmA to support the analysis of collaboration through high-level constructs such as attention has been demonstrated. This offers an opportunity to improve the analysis of non-verbal communication within team settings. For instance, Grover et al.^4^ explored non-verbal cues in pair programming, providing metrics for joint attention and body posture synchronization, which require further interpretation to assess their impact on communication^4^. Similarly, Kim et al.^7^, employed multimodal fusion techniques to enhance facial expression recognition by integrating audio and visual cues, highlighting the importance of multimodal approaches in understanding communication dynamics. In comparison, our study leverages audiovisual data to measure speaking time and attention distribution among team members, specifically within the context of agile methodologies such as planning poker. Our findings align with and extend the current state-of-the-art by offering a detailed analysis of how structured coordination techniques can influence communication patterns in agile software development teams.

## Data Availability

The datasets generated and/or analyzed during the current study are available from the corresponding author upon reasonable request.
